# Supplementary material for: Tuning strand displacement kinetics enables programmable ZTP riboswitch dynamic range in vivo
Source: Nucleic Acids Res. 2023 Mar 2;51(6):2891–903. doi: 10.1093/nar/gkad110 (PMC10085676; doi:10.1093/nar/gkad110)
Supplement: gkad110_Supplemental_Files [file gkad110_supplemental_files.zip › Bushhouse_ZTP_StrandDisplacement_SI_REVISED.pdf]

Supplementary Information for: Tuning Strand Displacement Kinetics Enables Programmable ZTP Riboswitch Dynamic Range *in vivo*

David Z. Bushhouse[1,2] and Julius B. Lucks[1,2,3,4,5,6] *

[1] Interdisciplinary Biological Sciences Graduate Program, Northwestern University, Evanston, Illinois 60208, USA
[2] Center for Synthetic Biology, Northwestern University, Evanston, Illinois 60208, USA
[3] Department of Chemical and Biological Engineering, Northwestern University, Evanston, Illinois 60208, USA
[4] Center for Water Research, Northwestern University, Evanston, Illinois 60208, USA
[5] Center for Engineering Sustainability and Resilience, Northwestern University, Evanston, Illinois 60208, USA
[6] International Institute for Nanotechnology, Northwestern University, Evanston, Illinois 60208, USA

* To whom correspondence should be addressed. Tel: 1-847-467-2943; Email: jblucks@northwestern.edu

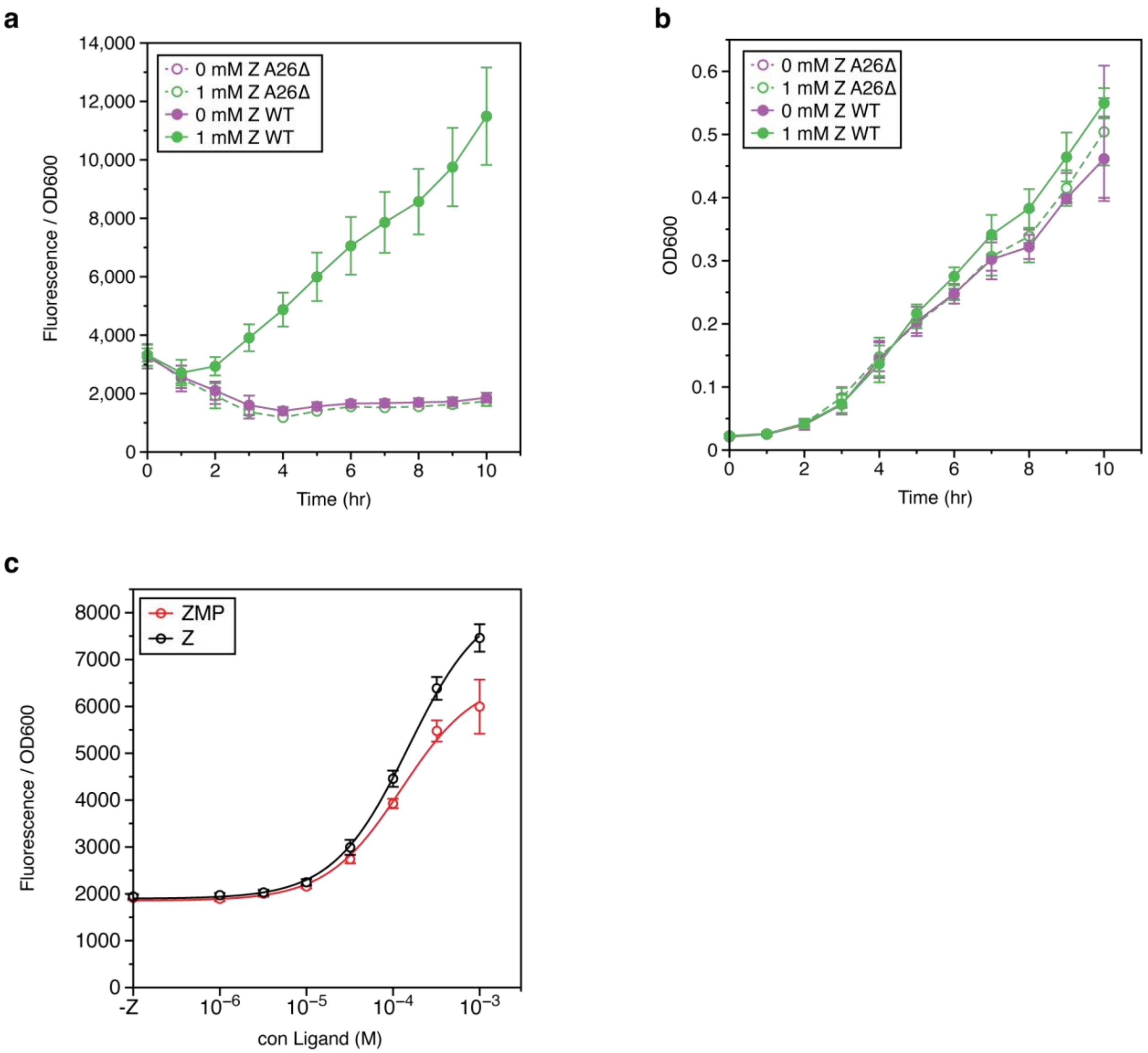

Supplementary Figure 1. Additional validation for *in vivo* reporter assay. (A) Time-course measurements of GFP fluorescence under the regulation of the *Cbe pfl* ZTP riboswitch show that by 6 hrs robust dynamic range is observed between the induced (1 mM Z) and uninduced (0 mM Z) subcultures. (B) Time-course measurements of subculture optical density shows that Z-induction has little effect on growth rate. (C) Dose-response curve at 6 hrs comparing response when the *Cbe pfl* ZTP reporter cassette is induced with Z or its monophosphorylated analog ZMP. Both curves show similar sensitivity and dynamic range, validating the use of Z in subsequent experiments. Data points represent averages from three experimental replicates, each performed with triplicate biological replicates for a total of nine data points (n=9), with error bars representing standard deviation.

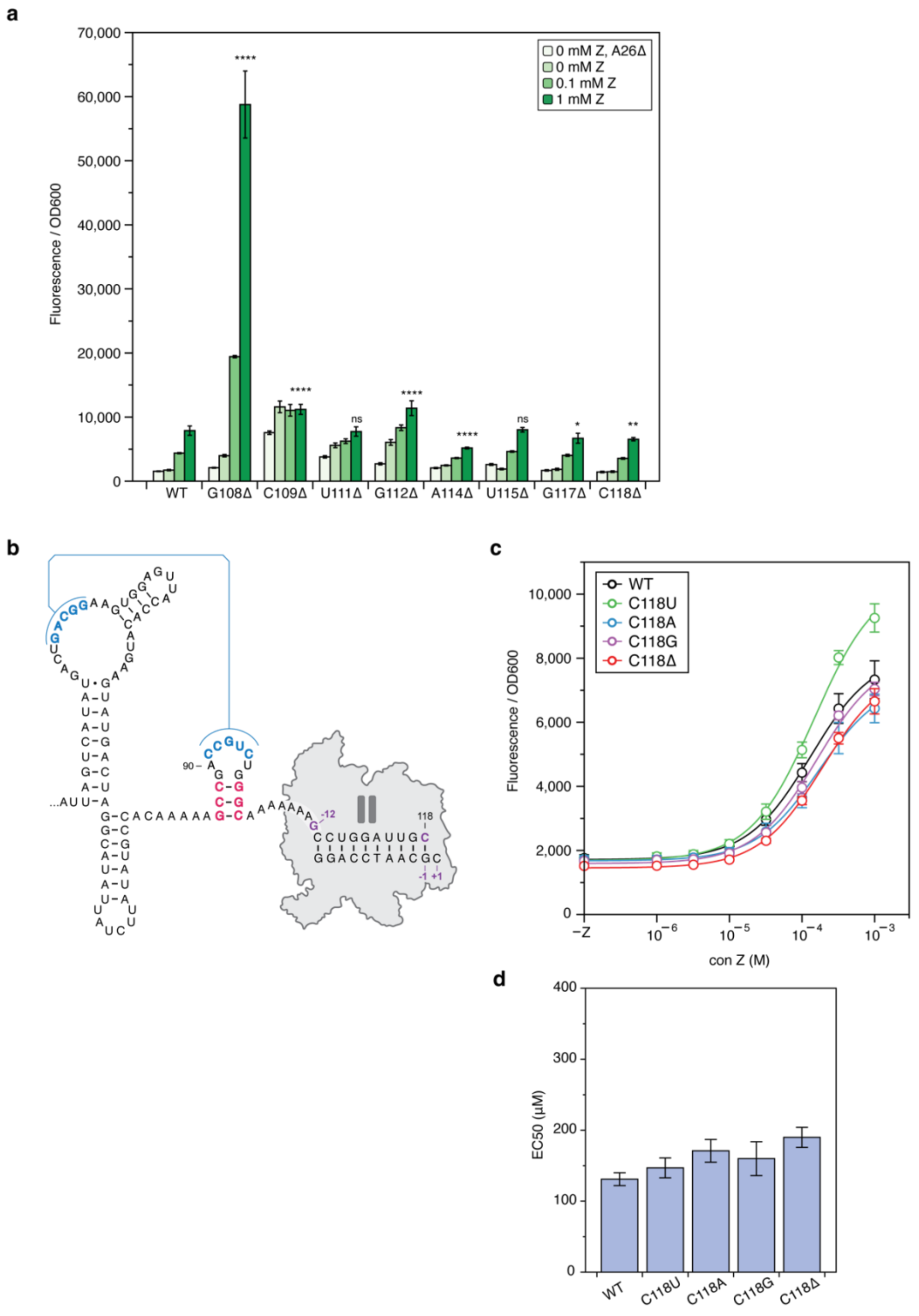

Supplementary Figure 2. Noncomplementary elements mediate tuning of dynamic range. (A) Deletion scan of the invader shows position-dependent effects of deletion mutations on ON-

state fluorescence. (B) Structure diagram of the C118 pause site 2. The ligand-binding competent aptamer is able to form. (C) Mutations designed to disrupt the C118 RNAP pause site 2 do not result in large changes to dynamic range. Data was fit according to a sigmoidal shape (see Methods). (D) EC50 values from the fits in (C) showing little change in riboswitch sensitivity from these variants. Bars in (A), and points in (C) represent averages from three experimental replicates, each performed with triplicate biological replicates for a total of nine data points (n=9), with error bars representing standard deviation. Bars in (D) represent values for the parameter EC50 (For equation see Methods), with error bars indicating standard error of the fit parameter. T-test significance thresholds in (A) are for pairwise (two-tail, heteroscedastic) tests between the 1mM and 0mM Z conditions, with values prior to Bonferroni correction of: * = 0.05, ** = 0.01, *** = 0.001, **** = 0.0001, ns = not significant.

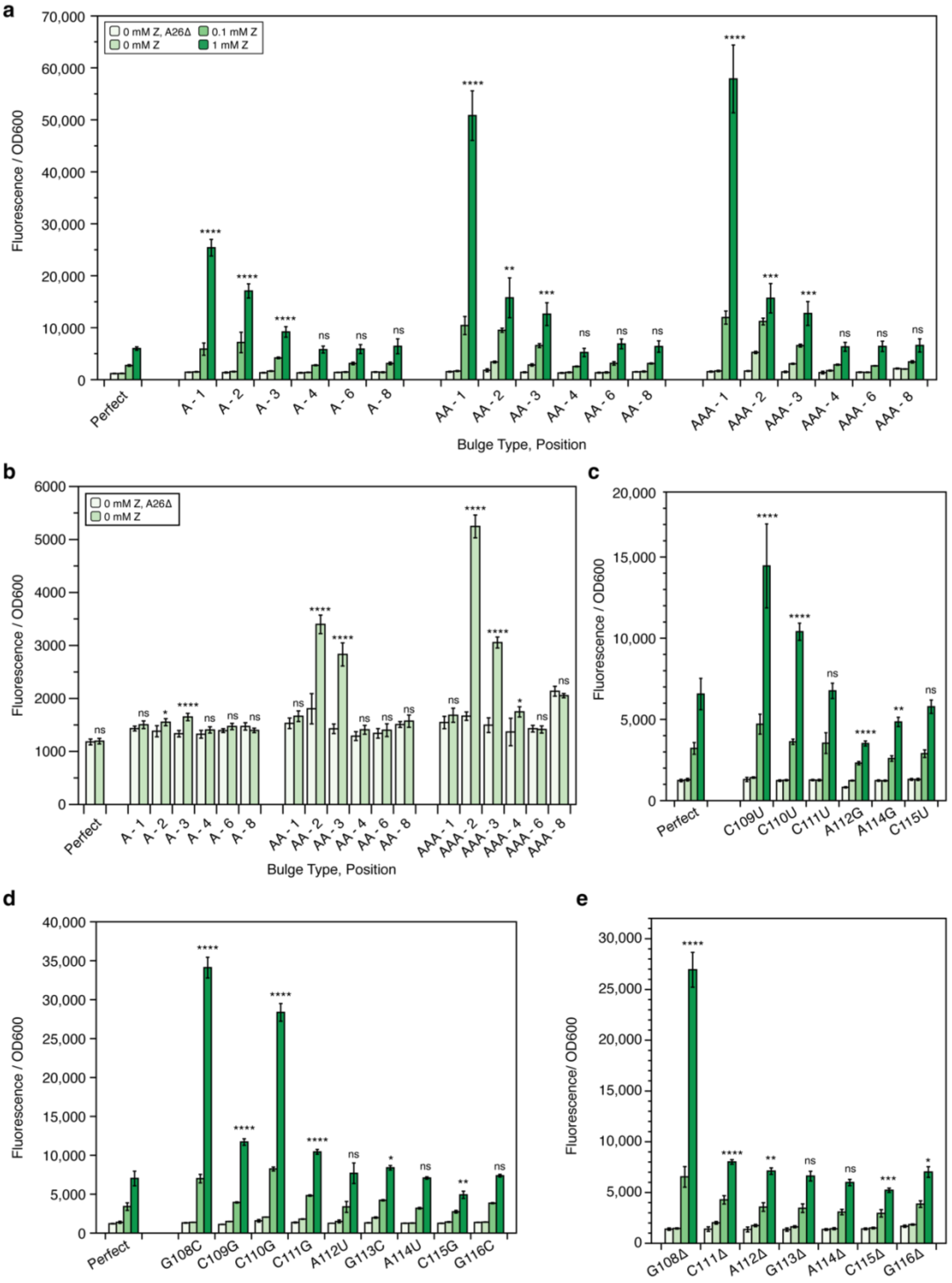

Supplementary Figure 3. Fine-grained mutational analysis of invader reveals rules underlying control of dynamic range. (A) Bulge scans of the perfectly complementary invader demonstrate position-dependent effect of noncomplementary elements on riboswitch dynamic

range. (B) A subset of the data from (A) is replotted to highlight differences in uninduced gene expression between the PK-incompetent background (A26Δ) and the PK-competent unmutated background. Larger kinetic barriers at positions 2 and 3 especially result in PK-dependent increases in leak. Mutational scans interrogating the effect of (C) wobble pairs, (D) mismatches, and (E) deletions demonstrate position-dependent effects of these noncomplementary elements on riboswitch dynamic range. Bars represent averages from three experimental replicates, each performed with triplicate biological replicates for a total of nine data points (n=9), with error bars representing standard deviation. T-test significance thresholds are for pairwise (two-tail, heteroscedastic) tests between ON-state (1 mM ZTP) FL/OD (A, C, D, E) or OFF-state (0 mM ZTP) FL/OD (B) with threshold values prior to Bonferroni correction of: * = 0.05, ** = 0.01, *** = 0.001, **** = 0.0001, ns = not significant.

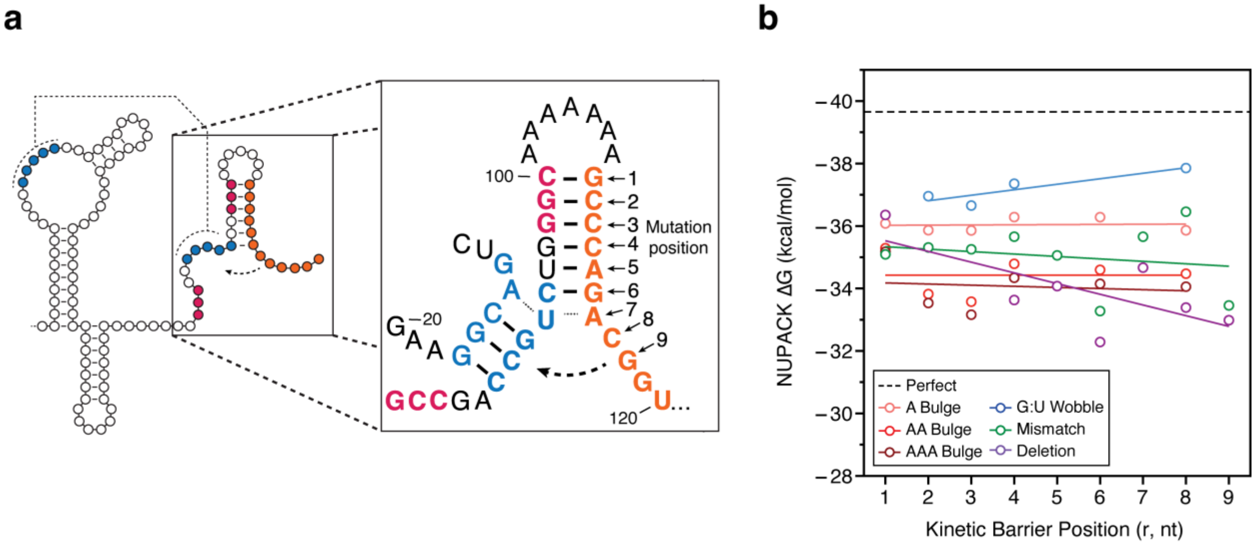

Supplementary Figure 4. NUPACK predicted conformational free energy of kinetic barrier mutants. (A) Diagram of the strand displacement process during terminator formation in the context of the perfectly paired terminator (Fig. 4A). (B) The predicted ΔG of the *Cbe* terminator hairpin (nt 85-124) carrying the specified kinetic barrier mutation. Structure prediction was performed using the NUPACK 4.0.0 default RNA model at 37°C.

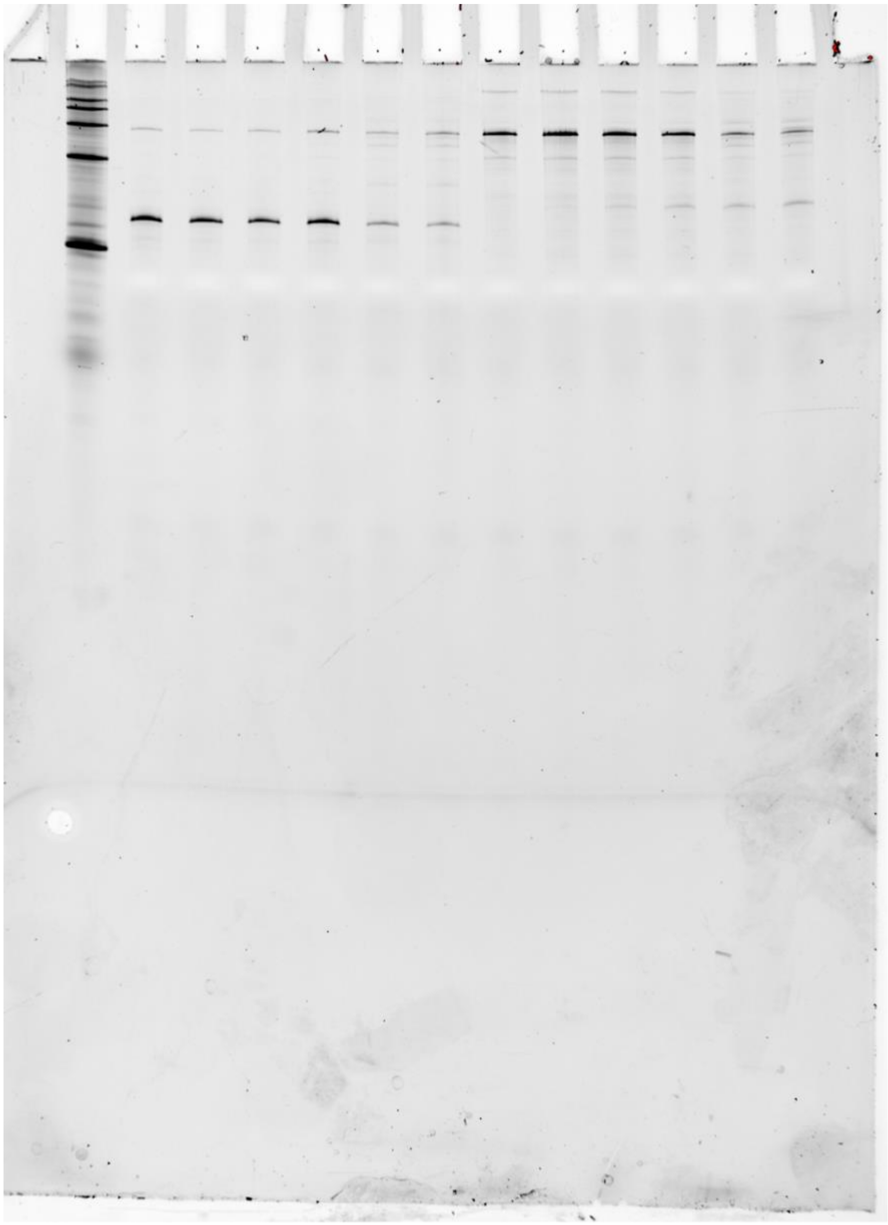

Supplementary Figure 5. Figure 5B Source Gel 1

1. RNA Century™-Plus Ladder (Invitrogen™)
2. *Cbe* wildtype, 0 µM Z, Replicate 1
3. *Cbe* wildtype, 10 µM Z, Replicate 1
4. *Cbe* wildtype, 32 µM Z, Replicate 1
5. *Cbe* wildtype, 100 µM Z, Replicate 1
6. *Cbe* wildtype, 320 µM Z, Replicate 1
7. *Cbe* wildtype, 1000 µM Z, Replicate 1
8. *Cbe* Flip, 0 µM Z, Replicate 1
9. *Cbe* Flip, 10 µM Z, Replicate 1
10. *Cbe* Flip, 32 µM Z, Replicate 1
11. *Cbe* Flip, 100 µM Z, Replicate 1
12. *Cbe* Flip, 320 µM Z, Replicate 1
13. *Cbe* Flip, 1000 µM Z, Replicate 1

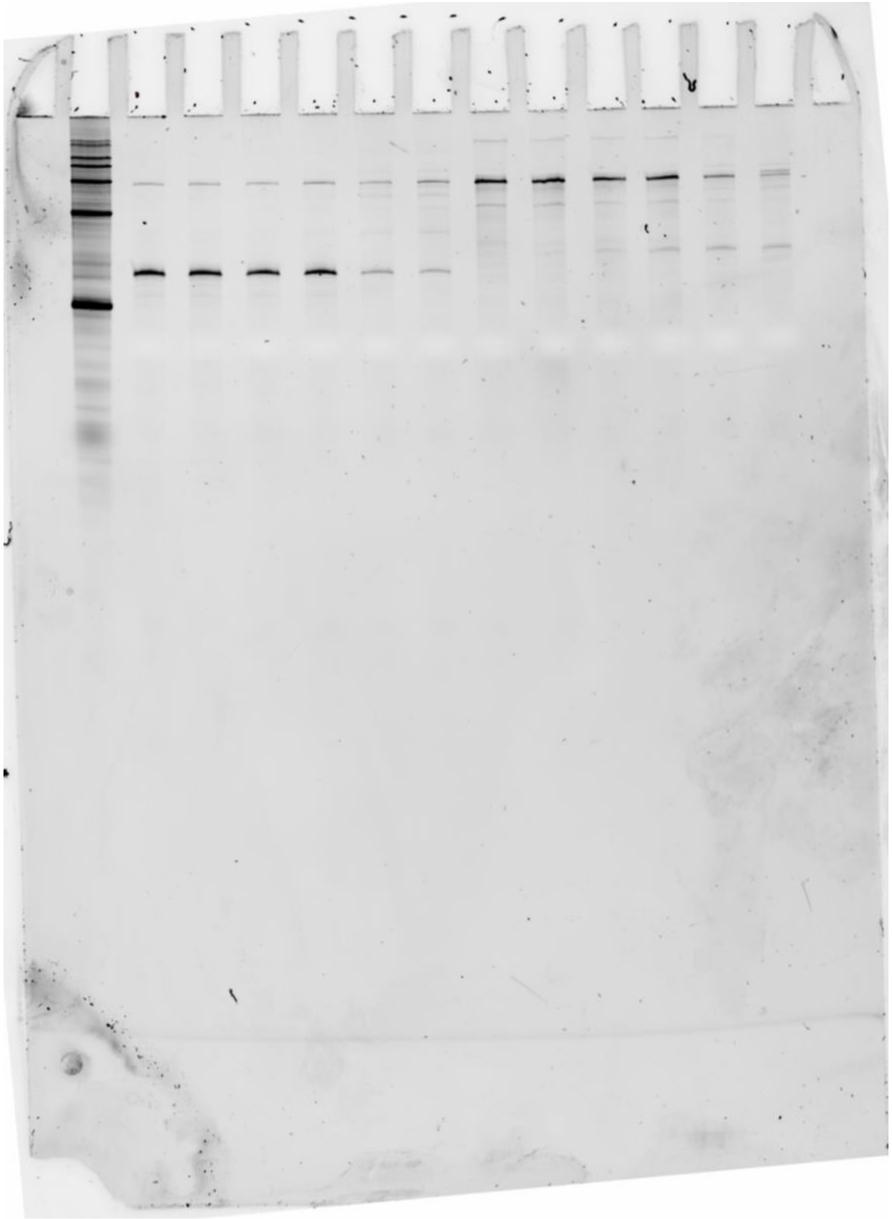

Supplementary Figure 6. Figure 5B Source Gel 2
1. RNA Century™-Plus Ladder (Invitrogen™)
2. *Cbe* wildtype, 0 µM Z, Replicate 2
3. *Cbe* wildtype, 10 µM Z, Replicate 2
4. *Cbe* wildtype, 32 µM Z, Replicate 2
5. *Cbe* wildtype, 100 µM Z, Replicate 2
6. *Cbe* wildtype, 320 µM Z, Replicate 2
7. *Cbe* wildtype, 1000 µM Z, Replicate 2
8. *Cbe* Flip, 0 µM Z, Replicate 2
9. *Cbe* Flip, 10 µM Z, Replicate 2
10. *Cbe* Flip, 32 µM Z, Replicate 2
11. *Cbe* Flip, 100 µM Z, Replicate 2
12. *Cbe* Flip, 320 µM Z, Replicate 2
13. *Cbe* Flip, 1000 µM Z, Replicate 2

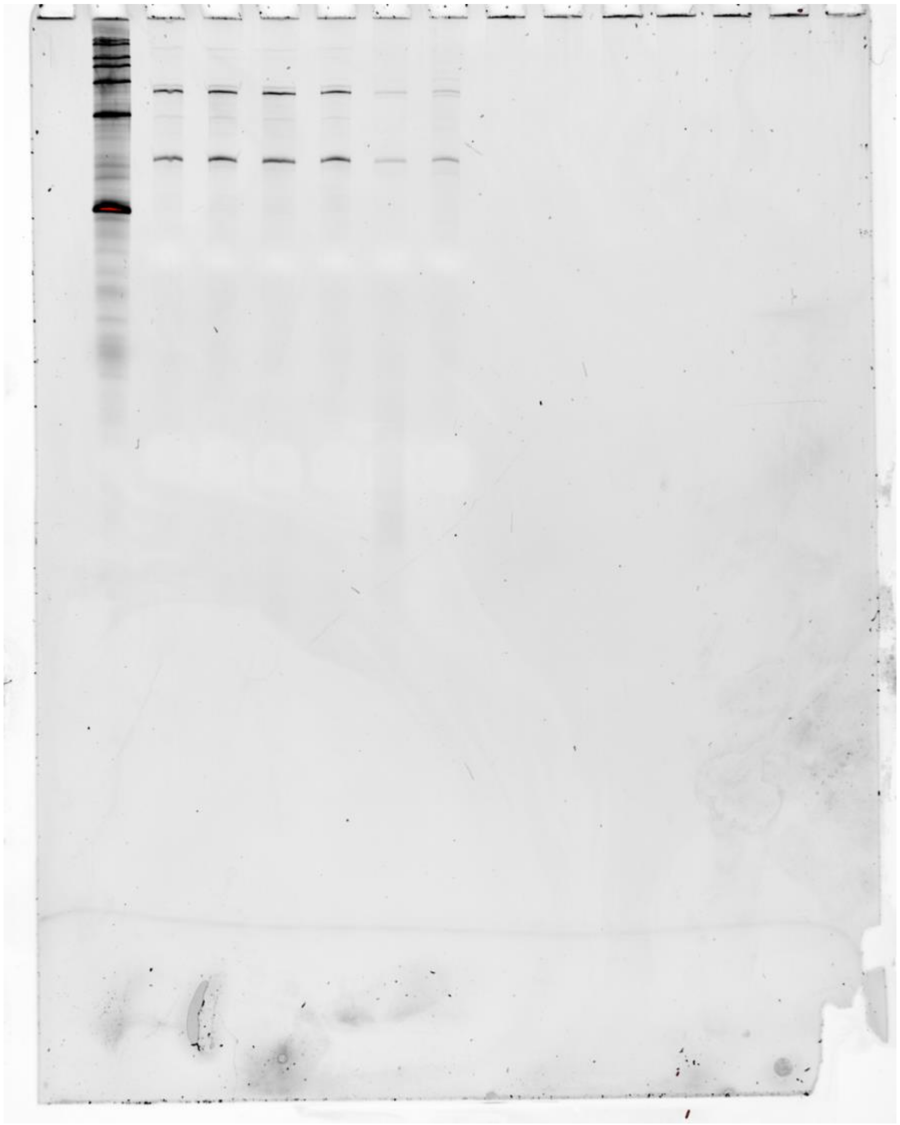

Supplementary Figure 7. Figure 5B Source Gel 3
1. RNA Century™-Plus Ladder (Invitrogen™)
2. *Cbe* AAA-Bulge Flip, 0 µM Z, Replicate 1
3. *Cbe* AAA-Bulge Flip, 10 µM Z, Replicate 1
4. *Cbe* AAA-Bulge Flip, 32 µM Z, Replicate 1
5. *Cbe* AAA-Bulge Flip, 100 µM Z, Replicate 1
6. *Cbe* AAA-Bulge Flip, 320 µM Z, Replicate 1
7. *Cbe* AAA-Bulge Flip, 1000 µM Z, Replicate 1

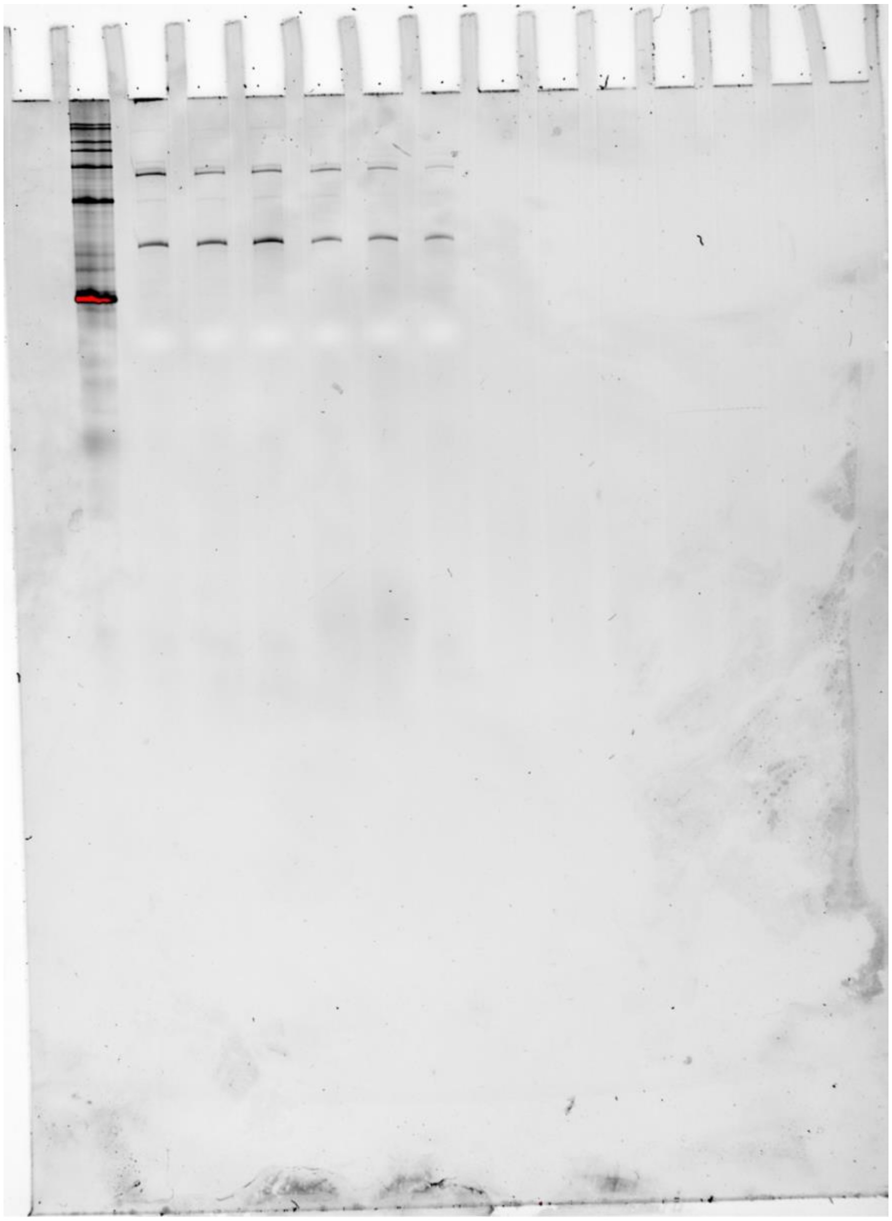

Supplementary Figure 8. Figure 5B Source Gel 4
1. RNA Century™-Plus Ladder (Invitrogen™)
2. *Cbe* AAA-Bulge Flip, 0 µM Z, Replicate 2
3. *Cbe* AAA-Bulge Flip, 10 µM Z, Replicate 2
4. *Cbe* AAA-Bulge Flip, 32 µM Z, Replicate 2
5. *Cbe* AAA-Bulge Flip, 100 µM Z, Replicate 2
6. *Cbe* AAA-Bulge Flip, 320 µM Z, Replicate 2
7. *Cbe* AAA-Bulge Flip, 1000 µM Z, Replicate 2

Species	Gene	GenBank Accession ID	Rfam Locus (RF01750)	Invader Seq	Reporter Construct
C. beijerinckii	pflA	CP000721.1	1211931…1212029	GCCTGGATTGCGTCGGC	pJBL3907
C. neopropionicum	pflA	LRVM01000003.1	315886…315803	GCCGACAATCCGGGC	pJBL6194
C. symbiosium	pflA	GL834314.1	200147…200067	GCCGGCAGTCCGGGC	pJBL6195
C. paraputrificum	pflB	MAPZ01000019.1	81979…81898	GCCGACAATCTGAGC	pJBL6196
C. saccharobutylicum	pflB	CP006721.1	1418877…1418954	GCCCAGACAGTCGGC	pJBL6215
C. beijerinckii	purH	CP000721.1	4111764…4111685	GTCCAGATAGTGGGC	pJBL6216
C. chromoreductans	purH	MZGT01000008.1	8289…8211	GCCCACTGTCCGGAC	pJBL6197
C. butyricum	purH	ACOM01000001.1	496623…496702	GTCCGGATAGGTGGGC	pJBL6199
C. bornimense	purH	HG917869.1	204603…204682	GTCCAGATGGTGGGC	pJBL6198

Supplementary Table 1. Source sequences used to generate chimeric riboswitches. For each invader sequence from the genus *Clostridium* that was used to generate a chimeric riboswitch, the species name, regulated gene (hyperlink to protein id), genome GenBank accession number, aptamer domain locus identified in Rfam, and invader sequence have been compiled. Sequences for indicated reporter constructs can be found in Supplemental Data File 1.
